# Characterization of PLA/PCL/Nano-Hydroxyapatite (nHA) Biocomposites Prepared via Cold Isostatic Pressing

**DOI:** 10.3390/polym15030559

**Published:** 2023-01-21

**Authors:** Solechan Solechan, Agus Suprihanto, Susilo Adi Widyanto, Joko Triyono, Deni Fajar Fitriyana, Januar Parlaungan Siregar, Tezara Cionita

**Affiliations:** 1 Department of Mechanical Engineering, Faculty of Engineering, Diponegoro University, Semarang 50275, Indonesia; 2 Department of Mechanical Engineering, Universitas Muhammadiyah Semarang, Kampus Kasipah, Semarang 50254, Indonesia; 3 Department of Mechanical Engineering, Sebelas Maret University, Surakarta 57126, Indonesia; 4 Department of Mechanical Engineering, Universitas Negeri Semarang, Kampus Sekaran, Gunungpati, Semarang 50229, Indonesia; 5 Faculty of Mechanical & Automotive Engineering Technology, Universiti Malaysia Pahang, Pekan 26600, Malaysia; 6 Faculty of Engineering and Quantity Surveying, INTI International University, Nilai 71800, Malaysia

**Keywords:** nano-hydroxyapatite, polylactic acid (PLA), polycaprolactone (PCL), biocomposites

## Abstract

Hydroxyapatite has the closest chemical composition to human bone. Despite this, the use of nano-hydroxyapatite (nHA) to produce biocomposite scaffolds from a mixture of polylactic acid (PLA) and polycaprolactone (PCL) using cold isostatic pressing has not been studied intensively. In this study, biocomposites were created employing nHA as an osteoconductive filler and a polymeric blend of PLA and PCL as a polymer matrix for prospective usage in the medical field. Cold isostatic pressing and subsequent sintering were used to create composites with different nHA concentrations that ranged from 0 to 30 weight percent. Using physical and mechanical characterization techniques such as Fourier transform infrared spectroscopy (FTIR), X-ray diffraction (XRD), scanning electron microscopy (SEM), and density, porosity, tensile, and flexural standard tests, it was determined how the nHA concentrations affected the biocomposite’s general properties. In this study, the presence of PLA, PCL, and nHA was well identified using FTIR, XRD, and SEM methods. The biocomposites with high nHA content showed intense bands for symmetric stretching and the asymmetric bending vibration of PO_4_^3−^. The incorporation of nHA into the polymeric blend matrix resulted in a rather irregular structure and the crystallization became more difficult. The addition of nHA improved the density and tensile and flexural strength of the PLA/PCL matrix (0% nHA). However, with increasing nHA content, the PLA/PCL/nHA biocomposites became more porous. In addition, the density, flexural strength, and tensile strength of the PLA/PCL/nHA biocomposites decreased with increasing nHA concentration. The PLA/PCL/nHA biocomposites with 10% nHA had the highest mechanical properties with a density of 1.39 g/cm^3^, a porosity of 1.93%, a flexural strength of 55.35 MPa, and a tensile strength of 30.68 MPa.

## 1. Introduction

Bone fractures caused by cancer, traffic accidents, bone tumors, or aging are incapable of self-healing. These bone fractures require interventional therapy with implants or bone grafts in order to heal and regenerate. Bone tissue engineering has garnered interest due to its inherent advantages for the healing of bone fractures.

Important components of bone tissue engineering are scaffolds that can provide dynamic circumstances for cell growth. Scaffolds must be biocompatible and biodegradable, possess appropriate mechanical qualities and pore sizes, and have pores that are well-connected [1,2,3]. The success of an implanted biomaterial is determined by a number of criteria, including its shape and structural characterization, durability, mechanical loading, property of implant material, location of the implanted site, and host reaction [3]. The standard treatment for bone repair is autografting, but it has a number of drawbacks, including limited tissue availability, discomfort for the patient, morbidity at the donor site, the need for a second procedure, challenges in fabricating an anatomically shaped graft, and a failure rate of up to 50% for some sites. The creation of implants and scaffolds was urgently needed in order to address these drawbacks. Thus, the engineered scaffolds’ goal is to rebuild bone tissue rather than just replace it [2].

Moreover, internal fixation devices made from metal may be utilized for the repair of bone fractures. Metallic materials such as stainless steel, CoCr alloys, Ti, and Ti alloys have a lot of good properties that mean they are utilized for bone implants. These properties include high fracture toughness, corrosion resistance, high strength, high hardness, and biocompatibility. However, the inadequate interfacial adhesion between metallic implants and tissue or bone results in the creation of a non-adhesive layer and movement at the implant tissue interface. This results in the failure of using metal implants to treat bone fractures. Furthermore, metallic implants have a substantially greater modulus of elasticity than bone, which can result in stress shielding. Consequently, osteoporosis, osteolysis, and secondary fractures will occur. During the implantation process, toxic effects induced by ions released from metallic implants represent a major problem [4,5,6,7,8,9,10]. The use of metal implants requires a second surgical procedure for implant removal, which can increase the cost of treatment [11]. This has prompted researchers to find substitutes for metal implants using other materials such as polymers, ceramics, and composites.

Presently, only a few polymer biomaterials are now available that are non-toxic, absorbable, and FDA-approved for use as scaffolding materials in medical applications. Among these, biomaterials with strong biocompatibility and biodegradability include polylactic acid (PLA) and polycaprolactone (PCL) [2,12]. PLA has a glass transition temperature (Tg) between 50 and 80 °C and a melting temperature (Tm) between 130 and 180 °C [13]. PCL has good solubility with other polymers, minimal viscosity, and hydrophobic characteristics, in which the molecular weight and crystallinity level affect the physical and mechanical characteristics. While the Tm ranges from 50 to 60 °C, the glass transition temperature (Tg) of PCL is roughly 60 °C. Take into account that PCL has a high crystallinity level (between 30 and 60%). However, the main limitations of PLA and PCL are their poor mechanical strength and low cell affinity limiting their application as bone scaffold materials [2,12].

PLA is a brittle material that degrades quickly in body fluids [14]. Therefore, the addition of PCL to PLA is used to minimize brittleness and extend the degradation time of the PLA. Subsequently, PLA and PCL are blended to make copolymers that have good qualities for tissue engineering, for instance, being biocompatible, biodegradable, and non-toxic [13,15,16]. Solechan et al. performed scaffold fabrication utilizing distinct PLA/PCL mixtures and observed the effects of PLA/PCL concentrations on their physical and mechanical properties. By increasing PLA content, the PLA/PCL blend porosity reduced, resulting in enhanced density and flexural strength [17]. An ideal scaffold ought to be osteoconductive (enabling pluripotent cells to develop into osteoblasts and supporting the proliferation of cells and capillaries to create bone), biocompatible, and biodegradable, as well as possess the proper biological qualities and mechanical strength [18]. To obtain the needed qualities, the incorporation of hydroxyapatite (HA) is able to overcome the hydrophobicity of the PLA/PCL blend, increase its mechanical properties, and stimulate osteoconduction, as well as osseointegration in the implanted scaffold [2,19,20].

Hydroxyapatite (HA) is known as the most common mineral found in bones and teeth. Because HA chemicals account for approximately 65% of bone, they are an interesting candidate for a synthetic bone composite. HA is a bioactive ceramic material that is extensively utilized in various biomedical applications, primarily as orthopedic implant materials and in the creation of dentistry materials [21,22,23].

Pijamit et al. utilize PLA/PCL/HA to be the biocomposite material for manufacturing 3D printing filaments. HA in the PLA/PCL/15HA mixture is used for producing the greatest compressive strength (82.72 ± 1.76 MPa). Furthermore, it was observed that HA also provided higher bone cell proliferation [16]. Fabrication of composite scaffolds made from PLA/PCL/HA by indirect 3D printing was studied by Hassanajili et al. According to their research, the scaffold with PLA/PCL 70/30 *w*/*w* and 35% HA had better osteoinduction, viability, and biocompatibility qualities [19]. Fitriyana et al. studied the effect of using HA on the mechanical, physical, and degradation properties of the composite materials using a matrix of PLA/PCL (80 wt%/20 wt%). According to their findings, the mechanical characteristics of the biocomposite got better as the HA concentration rose. However, the biocomposite degrades more quickly the greater the quantity of added HA content [20].

The use of additive manufacturing techniques to create scaffolds has been the subject of numerous investigations. The benefits of the additive manufacturing-based process for scaffold fabrication include closeness to the final dimensions, precision, and the capability of generating complex geometries, as well as low processing costs. The disadvantages that come along with this method are limited product size, relatively small dimensions, poor mechanical properties, the requirement of post-processing, which is expensive and time-consuming, residual stress, high surface roughness, frequently clogged nozzles, clumping, and the presence of delamination of the layers on the final product [24,25,26].

To overcome these problems, this research uses the cold isostatic pressing method to make biocomposites from PLA/PCL/nHA as a scaffold material. Prior to machining or sintering, powdered materials can be compacted via cold isostatic pressing to create a solid, uniform mass. The main advantage of cold isostatic pressing is the ability to produce products with more complex shapes. In addition, distortion and cracking due to non-uniform stresses are greatly reduced [27,28,29]. According to an investigation conducted by Abdallah et al., the use of the cold isostatic pressing method could improve the density, hardness, tensile strength, impact resistance, and ductility of the 93%W4.9%Ni-2.1%Fe alloy [30]. Cold isostatic pressing (CIP) has been widely used as an efficient processing process for the compaction of metal and ceramic powders. Compared to uniaxial pressing, CIP compression produces samples with a greater relative density, superior mechanical qualities, and a more uniform microstructure. According to some reports, after the CIP process, the ceramic powder can be compressed to a maximum relative density of 70%. After being subjected to high CIP pressure and sintering, the nanoparticles yield a relative density of up to 99.99 percent or more [31]. In the field of medical implants, porous metal structures are commonly utilized. By controlling the CIP and sintering process parameters, evenly porous metal components can be manufactured. Controlling porosity in CIP components necessitates a combination of parameters, including powder qualities, tool design, CIP process parameters, thermal processing conditions, and ingredient density throughout the process [32,33,34].

Al Bakri et al. found that the pressing process also affects the characteristics of the zirconia toughness alumina (ZTA) composite that is manufactured. CIP provides superior mechanical qualities compared to uniaxial pressing. Compared to the uniaxial pressing method, composites compacted using the CIP method exhibit superior characteristics at lower sintered temperatures [34]. The processes involved in the production of manganates are significantly affected by cold isostatic pressing. It is demonstrated that the compacting pressure has a bigger effect on the rate of a chemical reaction than on crystallization.

The results suggest that high hydrostatic pressures can be employed to reduce synthesis temperature and generate nanostructured ceramics and manganates with predetermined oxygen nonstoichiometry. The cold isostatic pressing affects manganate synthesis and increases the contact area, which mechanically activates the grains and amplifies the solid-phase sintering processes [35]. Akimov et al. showed that CIP has a big effect on the physical properties of many powders, such as stabilized zirconium dioxide, α-phase alumina powder, hydride-forming intermetallics LaNi_2.5_Co_2.4_A_l0.1_ and LaNi_5_, and manganese powder. In their research, they utilized the isostatic cold pressure technique to generate materials containing a mixture of amorphous boron powder, crystalline aluminum, and LaB_6_–TiB_2_ composites under 0.6 GPa of pressure and sintering at 1000 °C [36]. The decrease in crystallization temperature may be due to the fact that the CIP gives rise to crystallization nuclei. The sinterability and mechanical properties of green bodies subjected to cold isostatic pressing are significantly enhanced [37].

Cold isostatic pressing has not been intensively investigated as a method for producing scaffolds from PLA, PCL, or nHA biocomposites. This research was conducted to determine the effect of the concentration of HA used on the physical and mechanical properties of biocomposites with a PLA/PCL matrix prepared using the cold isostatic pressure method.

## 2. Materials and Methods

The properties of the polycaprolactone (PCL) and polylactic acid (PLA) used in this study are shown in Table 1. Meanwhile, nano-hydroxyapatite (n-HA) with particle size < 200 nm with 502.31 g/mol of molecular weight was obtained from Sigma-Aldrich Pte Ltd., Pasir Panjang, Singapore [38]. The PLA and PCL compositions used in this study were 80% and 20%, respectively.

The percentages of nHA composition used in this study were 0, 10, 20, and 30 wt%. The PLA/PCL/nHA biocomposite formulation is presented in Table 2. In a laboratory ball mill, PCL, PLA, and nHA were blended for two hours at 80 revolutions per min (Bexco; Haryana, India). The finished mixture was then added to a mold of stainless steel 304 with 17 mm diameter and 3 mm thickness. The compacting procedure occurred afterward, which generated a green body under a pressure of 40 MPa. Based on the reference, at a pressure of 40 MPa, there is a tangential contact between HA particles, as evidenced by the good grain bonding found in sintered particles. Moreover, the relative density increases by increasing uniaxial pressure between 10 and 40 MPa. At pressures between 40 and 190 MPa, the relative density tends to be constant [39]. Increased pressure during hot compression resulted in an increased melt flow index (MFI), crystallinity, density, ultimate tensile strength (UTS), and Young’s modulus of the PP-HA biocomposite. This happened because the mechanical bonding and surface locking of the HA and PP particles in the composite increased with increasing pressure, resulting in better mechanical properties and impact resistance [40].

The sintering technique was then performed on the green body formed at 150 °C for 2 h using a digital drying oven (D1570, made in Taiwan).

The developed PLA/PCL/nHA biocomposites were evaluated using FTIR, X-ray diffraction, scanning electron microscopy (SEM), and density, porosity, tensile, and flexural techniques.

In the PLA/PCL/nHA biocomposite, the functional groups constituting the material as well as the orientation of the molecular chain were identified using the Fourier transform infrared (FTIR) technique [41]. The functional groups in the PLA/PCL/nHA biocomposite were identified using a PerkinElmer Spectrum IR Version 10.6.1 spectrophotometer (USA). Aside from this, each spectrum was recorded in the range of 400 cm^−1^ to 4000 cm^−1^. X-ray diffraction (XRD) analysis was a non-destructive technique for investigating crystalline materials that was used to identify the crystalline phases in a material by looking at its crystal structure [42]. The PLA/PCL/HA biocomposites samples were identified by X-ray diffraction using a Shimadzu XRD-7000 diffractometer at 40 kV with a current of 30 mA and Cu K radiation (λ = 0.15406 nm). Diffractograms were obtained using a scanning rate of 1°/min between (2θ) 10° and 90°. This was followed with a step of 0.02. Furthermore, using a scanning electron microscope (SEM) and energy dispersive X-ray spectroscopy (EDX) (JSM-6510, JEOL, Japan) at an accelerating voltage of 15 kV, the surface morphology and elemental composition of the PLA/PCL/HA biocomposites were examined [43]. To make composition photos, topography images, and shadow images, a high-sensitivity backscattered electron detector was mounted on the bottom of the objective lens.

The actual density, theoretical density, and void volume (%) of PLA/PCL/nHA biocomposites were determined using studies by Satapathy et al. (2017) [44] and Taib et al. (2018) [45]. Flexural tests on PLA/PCL/nHA biocomposites were carried out based on the American Society for Testing and Materials (ASTM) number D790-17 to assess flexural strength. These properties were measured using the three-point bending test on a rectangular-shaped sample with a dimension of 127 mm × 12.7 mm × 3 mm. The crosshead speed used in the flexural test was 2 mm/min at room temperature.

Tensile strength was measured using PLA/PCL/nHA biocomposites specimens in accordance with the American Society for Testing and Materials (ASTM) D3039. In this study, the specimens for the tensile test were rectangular in shape, with dimensions of 250 mm × 25 mm × 3 mm. Aluminum tabs were fastened at the ends of the specimen to provide proper grip, prevent gripping damage, and ensure deep failure at the gauge length. A 50 kN load cell and a clip-on-type MTS extensometer with a gauge length of 25 mm were used to measure load and strain. A loading rate of 2 mm/min was used for testing. The flexural and tensile tests in this study used the HT-2402 Series Computer Universal Testing Machine from Hung Ta Instrument Co., Ltd., Sammutprakarn, Thailand, with five replications, and the average data were analyzed.

## 3. Results and Discussion

**Figure 1:**
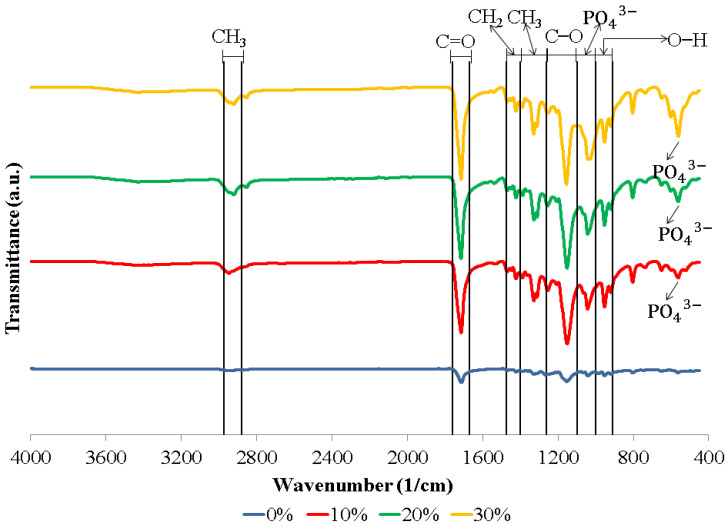
FTIR spectra of biocomposites with various concentrations of nHA.

Figure 1 shows the FTIR spectra of the biocomposite specimens. The biocomposite obtained in this study formed functional groups with almost similar wavenumbers. The appearance of C–O, C=O, and CH_3_ peaks in the biocomposite specimens demonstrate that polylactic acid (PLA) was present. Meanwhile, the C–O, C=O, and CH_2_ peaks indicate the presence of polycaprolactone (PCL) in the biocomposite specimens. The existence of PO_4_^3−^ and O–H peaks in the biocomposite specimens indicates that nano-hydroxyapatite (nHA) is present [17,46,47]. The hydroxyapatite (HA) spectrum demonstrates that the PO_4_^3−^ stretch band is around 1156–1000 cm^−1^ and the O–H bend is in the range of 950–910 cm^−1^. Following the O–H bend, a band at 631 cm^−1^ showing the extension of the hydroxyl group (OH-) in nHA was also detected in all biocomposite samples. In this study, all of the biocomposite specimens showed a weak O–H stretch in the range of 3160–3640 cm^−1^ [46,47,48,49,50]. The results of the FTIR test in this study found that a peak between 1440 and 1475 cm^−1^ indicates a CH_2_ asymmetric stretch. A peak between 2880 and 2975 cm^−1^ shows a CH_3_ symmetrical stretch [51,52]. FTIR test results showed the presence of symmetrical bending of CH_3_ in specimens of 0, 10, 20, and 30% at peaks of 1373, 1335, 1332, and 1336 cm^−1^, respectively. Thus, the C=O and C–O stretches are measured at 1760–1670 cm^−1^ and 1100–1000 cm^−1^ [17,46].

The biocomposite specimens in this investigation demonstrated all of the peaks that were representative of nHA, PCL, and PLA. Furthermore, the findings of this investigation revealed no novel peaks in the spectrum. This suggests that the two polymers and nano-hydroxyapatite have a weak interaction and are fully incompatible. The results of this study are in accordance with the results of research conducted by Hassanajili et al., Shojaei et al., and Åkerlund et al. Based on the FTIR spectra, they found that there is a weak interaction between PLA, PCL, and HA, which indicates that they are completely incompatible. The carbonyl groups shifted, indicating that PLA, PCL, and HA interact with each other [19,46,47].

Figure 1 presents the FTIR spectrum of the biocomposite from PLA/PCL/nHA prepared via cold isostatic pressing. For the biocomposites obtained via isostatic cold pressing, the PCL, PLA, and nHA bands were well identified, with different band intensities being affected by the concentration of nHA used. Furthermore, biocomposites containing a high nHA content exhibited intense bands at 1047 and 551 cm^−1^, which were associated with the symmetric stretching vibration of PO_4_^3−^ and the asymmetric bending vibration of PO_4_^3−^. The findings in this study are similar to the results of research conducted by Bernardo et al. They made 3D filament from a biocomposite of PLA and HA. Their results showed that with a higher concentration of HA used, the FTIR spectra showed bands with stronger intensities at 1026 and 563 cm^−1^, representing a symmetric stretching vibration of PO_4_^3−^ and asymmetric bending vibration of PO_4_^3−^, respectively [53].

**Figure 2:**
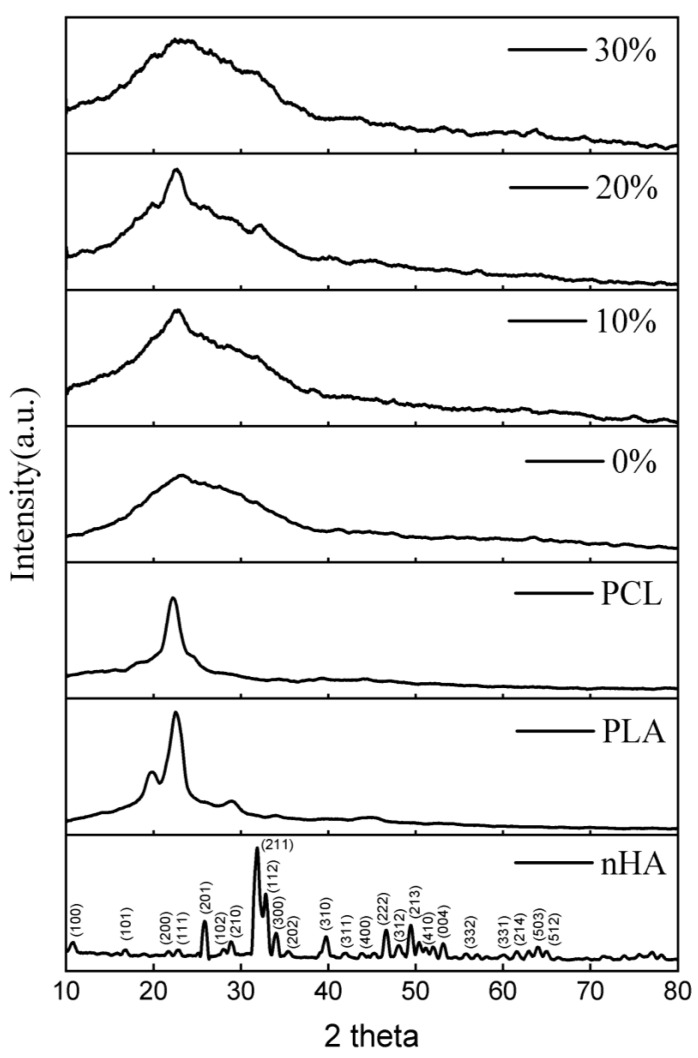
XRD patterns of the nHA, and PLA/PCL/HA biocomposites with various concentrations of nHA.

X-Ray diffraction (XRD) was utilized to identify the biocomposites’ crystallized phases (Figure 2) within the 2θ range from 10° to 80°. The 2 theta values of 10.820; 16.841; 22.902; 25.879; 28.966; 31.773; 32.196; 32.902; 34.048; 39.818; 43.804; 46.711; 48.103; 49.468; 50.493; 51.283; 52.100; 53.143; 55.879; 61.660; 64.078; and 65.031 reflect the nHA peak based on the JCPDS card number 09-0432 for the stoichiometric peak of HA. The XRD peaks produced in this study have similarities with the results of research conducted by Herliansyah et al. The results of their research show the XRD graph on HA from bovine (BHA) with 2θ at 21, 22, 25, 28, 31, 32, 34, 35, 39, 41, 43, 45, 46, 48, and 49° [54]. Peaks at 2 theta values of 19.76, 22.74, and 28.82 suggested the presence of PLA. The peak that indicates PCL is denoted by the 2 theta value of 22.42 [17,55,56].

The lack of crystal peaks in the PLA/PCL blend (0% nHA) suggests that an amorphous structure is generated. When PCL is incorporated into PLA, a disordered structure is formed, making crystallization more difficult [17].

In this investigation, the PLA/PCL/nHA biocomposite exhibited amorphous phase dominance, as evidenced by the broadening of peaks between 2 thetas 10.00° and 40.00° for all nHA content variations (10%, 20%, and 30%). This occurred because the integration of nHA into the PCL and PLA matrix resulted in a less homogeneous structure and essentially no crystalline phase formation. The reason for this is that the addition of HA interferes with the arrangement of the PLA and PCL molecules, preventing crystal formation [57]. In addition, the hydrogen bonds between nHA and PCL/PCL inhibit the orderly arrangement of PCL and PLA molecular chains, thereby reducing crystallinity [58]. This investigation’s findings are consistent with those reported by Pires et al. According to their research, the addition of 30 wt% glass prevented polymer matrix crystallization. Consequently, PCL–bioglass composites have a lower crystallinity than pure PCL. This behavior is the result of the interaction between the filler and matrix interfaces. At high concentrations, bioglass inhibits the movement of polymer molecules. This results in a less crystalline or amorphous polymer [59].

**Figure 3:**
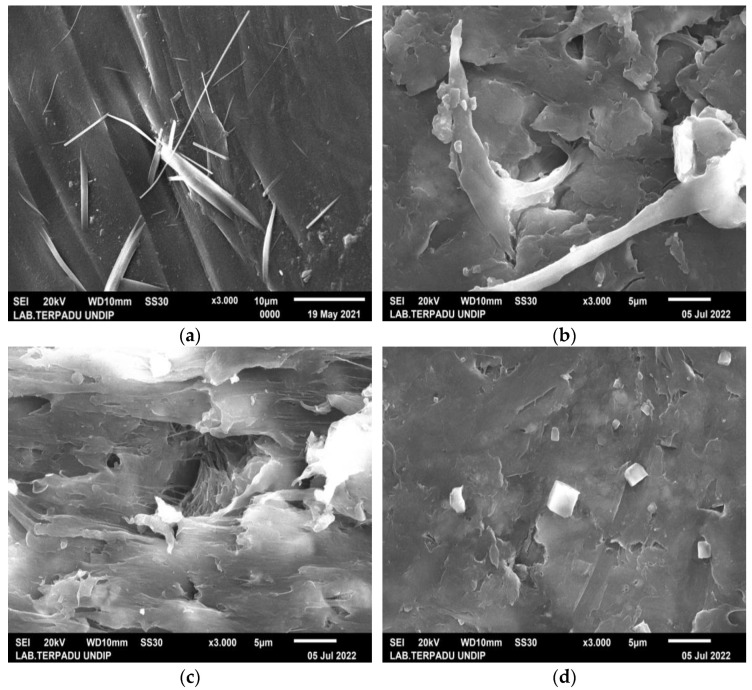
SEM images of PLA/PCL/nHA biocomposite specimens of (**a**) 0%; (**b**) 10%; (**c**) 20%; and (**d**) 30%, at 3000× magnification.

**Figure 4:**
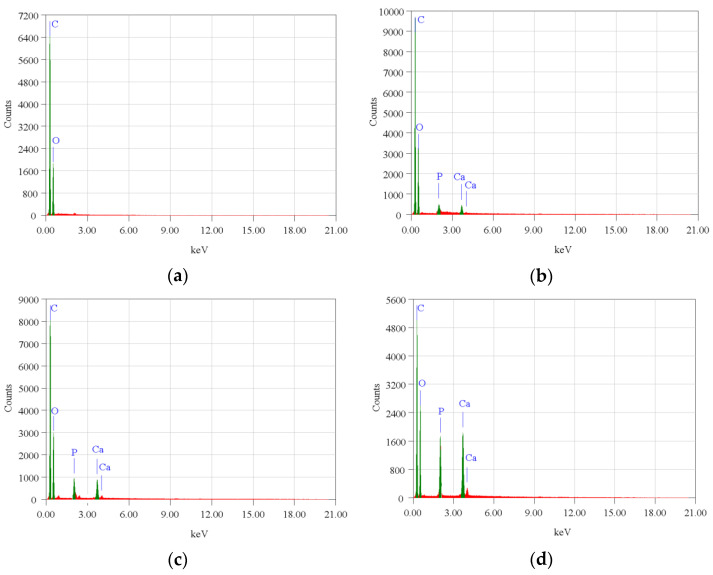
EDX profile of the PLA/PCL/nHA biocomposite specimens of (**a**) 0%; (**b**) 10%; (**c**) 20%; and (**d**) 30%.

The surface morphology of biocomposite samples has been examined using a scanning electron microscope (SEM). Figure 3 demonstrates that nHA was equally distributed throughout the biocomposite. The nHA (small white particles) is evenly distributed throughout the biocomposite. However, nHA agglomerations of diverse sizes were seen in biocomposites with higher nHA content (Figure 3b,c). This is because the biocomposite sample with a greater nHA content increases the surface energy between PCL and nHA, lowering the interfacial contact between PCL and nHA and causing the agglomeration of nHA particles in the polymer matrix [16,19]. The more nHA added resulted in higher Ca and P peaks on the EDX graph as shown in Figure 4.

However, in specimens with 0% nHA, the EDX graph only found C and O peaks. Aside from this, with more nHA added, the atomic (%) of Ca and P also increased (Table 3). The SEM and EDX test results support the XRD and FTIR test results which proved that nHA had been successfully incorporated into the PLA/PCL/nHA composite.

The studies of Cardoso et al. and Doyle et al. also showed similar results to this study. The agglomeration of nHA particles in the polymer matrix is caused by dispersion problems that occur during the mixing process in the raw materials [60,61]. According to Doyle et al., mixing nHA particles with chloroform can reduce agglomeration and produce a homogeneous dispersion of the nHA particles [50]. The unification of nHA into the polymer matrix not only improves the material’s bioactivity but also increases surface roughness, which has the ability to alter cell adhesion and proliferation. Furthermore, nHA addition to the biocomposite material will result in the production of a porous surface. Adequate porosity of appropriate sizes and linkages between pores improves cell infiltration, migration, vascularization, oxygen and nutrient flow, and waste material elimination [46,62].

**Figure 5:**
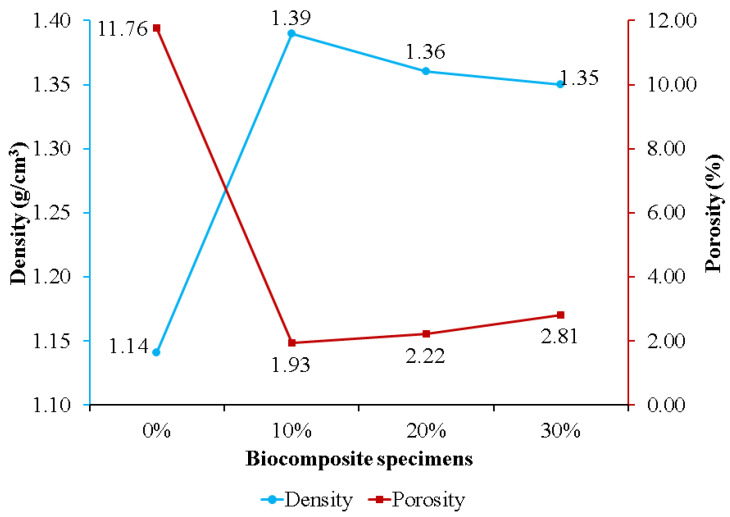
The density and porosity of PLA/PCL/nHA biocomposites.

Figure 5 exhibits the effect of nHA concentration on biocomposite specimen density and porosity. Specimens without nHA had the highest porosity and lowest density of 11.76% and 1.14 g/cm^3^, respectively. Adding nHA to the PLA/PCL matrix resulted in lower porosity than the sample without nHA (0%). This is because the nHA within the PLA/PCL matrix is strongly bound and is believed to be involved in the chemical changes that occur during biocomposite fabrication. During the process, nHA is adsorbed into the PLA/PCL matrix to fill voids and increases in density as the porosity of the biocomposite decreases [63]. The findings of this study are consistent with those of Kareem et al. and Kim et al. According to the findings of Kareem et al., adding 10% HA to the PLA matrix induced a decrease in porosity and an increase in density [64]. Meanwhile, Kim et al. found that adding 10% HA to the PCL matrix resulted in lower porosity than pure PCL porosity [65].

The porosity of the biocomposite specimens increased as the concentration of nHA used increased. This occurred because the addition of nHA inhibited sintering for the matrix (PLA/PCL), resulting in the formation of pores in the biocomposite specimens [66]. Furthermore, biocomposite specimens containing 30% nHA exhibited greater porosity than biocomposite specimens using 10% and 20% nHA. The increase in porosity in the biocomposite specimens was due to the greater density of nHA compared to the matrix (PLA/PCL). With constant total nHA and matrix quality, the volume decreases as density increases with the addition of more nHA, resulting in the formation of more pores following solid–liquid phase separation [67]. The findings in this study are in accordance with the findings in research conducted by Fang et al. [66] and Casadei et al. [67]. Their research showed that the HA concentration addition increased the porosity of the scaffold biocomposite made of PLLA and HA.

The density of biocomposite specimens decreases as porosity rises [17,68]. The density of biocomposites obtained, formed from PLA, PCL, and nHA, is displayed in Figure 5. Biocomposites with a 0% nHA concentration produced the lowest density, 1.14 g/cm^3^, while biocomposites with a 10% nHA concentration produced the highest density, 1.39 g/cm^3^. The density of the biocomposite decreases as the nHA content increases from 10% to 30%. This is due to the wettability and clustering of nHA as the reinforcement particles [69]. The biocomposite specimen produced in this research has a density between 1.1 and 1.3 g/cm^3^, which is nearly equivalent to that of human cortical bone density [70]. This explanation is consistent with Yousefpour et al.’s. In comparison with their results, increasing the HA concentration decreased the density of the Ce-TZP/Al_2_O_3_/HA bio-nanocomposite.

Furthermore, the density of the specimen with low HA is relatively identical to the density values noted in the theory. The increase in HA concentration resulted in the difference between the measured density and the theoretical density increasing [71].

**Figure 6:**
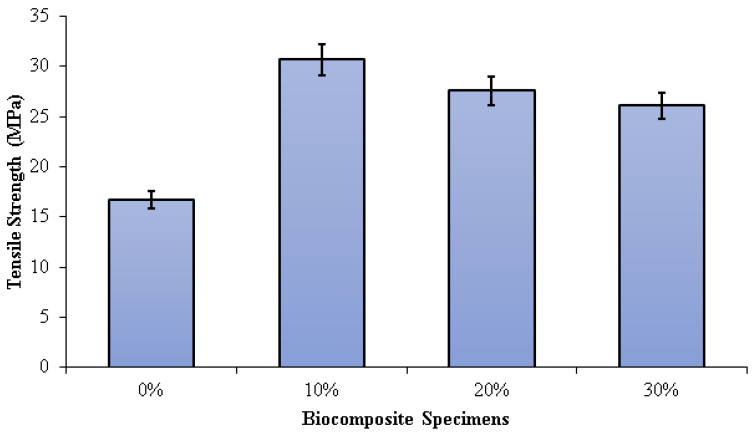
Tensile strength of PLA/PCL/nHA biocomposite specimens.

The tensile strength of the PLA/PCL matrix (0% nHA) increased with the addition of nHA (Figure 6). This is due to the density increase that occurs with the addition of nHA. As the density of biocomposites increases, the ultimate tensile strength increases. As the density increases, the compatibility of the nHA matrix with the PLA/PCL improves, and the strength of the composite increases. However, the sample without nHA (0%) has low density due to high porosity. Higher porosity concentrates stress and reduces load-bearing capacity, thus reducing the strength of the material. The higher the compatibility, the more effectively the nHA can transfer stress between the PLA/PCL matrix. Therefore, nHA has a better stress concentration and can withstand higher stresses when stretched or pulled before failure [72]. The tensile strengths of the specimens 0%, 10%, 20%, and 30% were 16.75 MPa, 30.68 MPa, 27.57 MPa, and 26.07 MPa, respectively. The highest tensile strength in this study was found in biocomposite specimens with an nHA concentration of 10%. This occurs as a result of well-dispersed nHA particles, thereby extending the fracture propagation path, absorbing some of the energy, and increasing plastic deformation.

Consequently, the surface fracture strength and energy of the biocomposite specimen increase. Furthermore, as the concentration of nHA rises, the size of the voids form when the polymer matrix disintegrates from the nHA particles. When this happens, significant cracks begin to occur. In addition, the increased agglomeration of nHA particles resulting from uneven dispersion reduces the biocomposite’s strength [73,74,75,76]. Aldabib et al. achieved the same findings in their study. Once the HA loading exceeded 5 wt%, the tensile strength declined. The use of a higher concentration of nHA resulted in agglomerations and an uneven distribution of nHA particles in the matrix. More agglomeration results in higher porosity. This phenomenon causes a reduction in the biocomposite’s density and tensile strength [73].

Dehestani et al. found that as the content of HA increased, the tensile strength and ductility of iron–hydroxyapatite composites dropped. The higher the HA content, the more unequal the HA particle dispersion in the Fe matrix and the lower the tensile strength of the biocomposite. The magnitude of the decrease in mechanical properties obviously depends not only on the content of HA but also on the distribution of HA dispersed in the Fe matrix and the particle size of the HA used [74]. According to research conducted by Ma et al., increasing the amount of HA from 0% to 40% increased the elastic modulus and decreased the tensile strength.

When the HA concentration is 30 percent or less, the tensile strength of the HA/PEEK composite is greater than that of cortical bone (50 MPa). The 40% HA/PEEK composite’s tensile strength, however, was less than 50 MPa, rendering it incompatible with cortical bone [61]. According to research by Kang et al., the elastic modulus of the composite increased from 2.36 GPa to 2.79 GPa as the HA content increased from 10% to 30%, while the tensile strength decreased from 95 MPa to 74 MPa. The homogeneity of various particle sizes and dispersions has a significant impact on mechanical properties. The 10% HA concentration improved dispersion in the PEEK matrix and enhanced the composite’s tensile strength [76].

**Figure 7:**
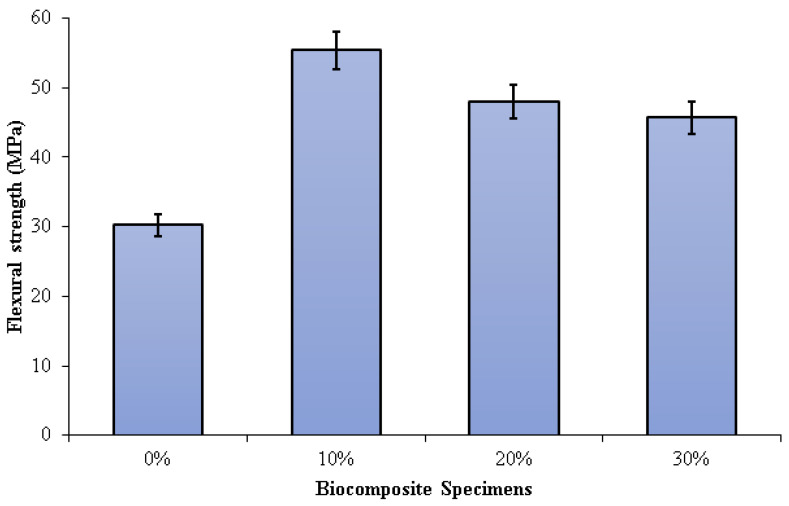
Flexural strength of PLA/PCL/nHA biocomposite specimens.

Figure 7 depicts the three-point bending test results on PLA/PCL/nHA biocomposites. The flexural strength of the PLA/PCL matrix was significantly enhanced by the addition of nHA (10%, 20%, and 30%). Increased adhesion between the PLA/PCL matrix and nHA results in greater stress transfer from the polymer matrix to nHA. The presence of nHA in the PLA/PCL blend facilitates the formation of a more rigid bond, which contributes to the improvement of flexural strength.

Flexural strengths of 0%, 10%, 20%, and 30% of specimens were 30.21 MPa, 55.35 MPa, 47.99 MPa, and 45.67 MPa, respectively. The highest flexural strength (55.35 MPa) was obtained in biocomposite specimens containing 10% nHA. The flexural strength of the biocomposite specimens decreases as the nHA concentration increases from 10% to 30%. A rise in nHA concentration results in significant nHA agglomeration in the matrix. Consequently, a propagating fracture may form as a result of stress concentration, which subsequently readily results in brittle failure. Furthermore, the addition of nHA with a concentration of 10% gave a suitable stiff phase in the matrix made of a mixture of PCL and PLA. The use of nHA with the right concentration can limit deformation and mobility in the matrix, resulting in high flexural strength [77].

Aldabib et al. explained that the homogeneous distribution of nHA particles within the biocomposite can be linked to the increased flexural strength of the biocomposite specimen at lower nHA concentrations. The flexural strength increases as the dispersion of nHA particles improves [73]. As previously explained, a higher concentration of nHA used results in an increase in the porosity of the biocomposite specimen. Due to this, mechanical properties such as tensile strength and flexural strength diminish as well as density. Thus, a higher concentration of nHA used makes a decrease in the flexural strength of the biocomposite specimen. Based on the study of Yadav et al., the cause of the decrease in flexural strength in dental restorative composite specimens with an inclination in nHAPs filler could be due to the presence of harder and stiffer ceramic particles resulting in brittle composites.

Furthermore, the higher concentration of nHA resulted in the formation of a lot of pore content in the composite specimen [78]. Comparably similar findings were reported by Nawang et al. It was discovered that increasing the amount of filler decreased flexural strength [79]. The liquid absorption and the amount of contraction stress decreased as the hydroxyapatite added to the polymer matrix was increased. Thus, the resulting bond strength and flexural strength of the composite decreased [80].

The investigation findings are aligned with Ferri et al.’s research. Their findings show that the higher the concentration of HA used, the lower the flexural strength of the PLA/HA composite. Flexural strength is reduced as a result of the biocomposites’ greater stiffness produced by the increased HA concentration. Furthermore, when HA concentration increases, particle aggregation becomes more possible, and the nucleating effect becomes less evident. As a result, the cracking probability is increased because HA aggregates perform as crack initiators [81]. According to Shyang et al. [67] and Bilic-Prcic et al. [68], an increase in hydroxyapatite concentrations results in a decrease in flexural strength.

## 4. Conclusions

The PLA/PCL/nHA biocomposites were successfully prepared via the cold isostatic pressing method. The biocomposites obtained via isostatic cold pressing, PCL, PLA, and nHA bands, were well identified using the FTIR test. The different band intensities are affected by the concentration of nHA used. The biocomposites with high nHA content showed intense bands at 1047 and 551 cm^−1^, which were associated with a symmetric stretching vibration of PO_4_^3−^ and asymmetric bending vibration of PO_4_^3−^, respectively. The PLA/PCL/nHA biocomposites lack crystal peaks, indicating that the resulting structure is amorphous. A broadening of the peak between 2 thetas of 10.00° and 40.00° on the XRD diffractogram indicated an increase in the distance between PLA layers. The spacing between PLA layers increased, indicating a more amorphous phase in these PLA/PCL/nHA biocomposites, whereas the addition of nHA led to a less uniform structure and made crystallization more difficult. The nHA (small white particles) was evenly distributed throughout the biocomposite. However, nHA agglomerations of diverse sizes were seen in biocomposites with higher nHA content.

The addition of nHA improves the density and tensile and flexural strength of the PLA/PCL matrix (0% nHA). However, by increasing nHA content, the PLA/PCL/nHA biocomposites became more porous. In addition, the density of the PLA/PCL/nHA biocomposites decreased linearly as the nHA concentration increased. The PLA/PCL/nHA biocomposites with 10 wt% nHA exhibited the highest density (1.39 g/cm^3^) and the smallest porosity (1.93%).

The flexural and tensile strength of the PLA/PCL/nHA biocomposite decreased with decreasing density. The tensile strength of biocomposite specimens decreased when the nHA concentration exceeded 10 wt% because the nHA particles were not well dispersed. Furthermore, the increase in the concentrations of nHA led to a decrease in flexural strength. Flexural strength is reduced as a result of the biocomposites’ greater stiffness produced by the increased nHA concentration. The highest tensile and flexural strength were found in PLA/PCL/nHA biocomposites with 10 wt% nHA, with a tensile and flexural strength of 30.68 MPa and 55.35 MPa, respectively.

## Figures and Tables

**Table 1:** The properties of the polycaprolactone (PCL) and polylactic acid (PLA) [17].

Specifications	PLA	PCL
Form	Powder	Pellet
Manufacturer	Reprapper Tech Co., Kowloon, Hong Kong	Solvay Interox Limited, Warrington, UK
Density (g/cm^3^)	1.24	1.1
Melting temperature (°C)	175–220	58–60
Grain size (µm)	5–10	-
Diameter (mm)	-	0.5

**Table 2:** Label and composition of biocomposite specimens.

Polymeric Blends	Ratio (*w*/*w*)	HA (wt%)	Specimen Codes
PLA/PCL	80/20	0	0%
PLA/PCL	80/20	10	10%
PLA/PCL	80/20	20	20%
PLA/PCL	80/20	30	30%

**Table 3:** Summary of EDX test results of PLA/PCL/nHA biocomposites.

Element	Atom (%)			
	0%	10%	20%	30%
C	75.73%	68.00%	67.77%	63.91%
O	24.27%	31.00%	30.39%	32.62%
Ca		0.76%	0.97%	1.88%
P		0.24%	0.87%	1.59%
Total	100%	100%	100.00%	100.00%

## Data Availability

Data are contained within the article.
